# Optimization of water distribution of network systems using the Harris Hawks optimization algorithm (Case study: Homashahr city)

**DOI:** 10.1016/j.mex.2020.100948

**Published:** 2020-06-04

**Authors:** Saeid Khalifeh, Saeid Akbarifard, Vahid Khalifeh, Ebrahim Zallaghi

**Affiliations:** a Faculty of Agriculture, Ferdowsi University of Mashhad, Mashhad, Iran; b Department of Hydrology and Water Resources, Faculty of Water Sciences Engineering, Shahid Chamran University of Ahvaz, Ahvaz, Iran; c Faculty of Civil, Sirjan University of Technology, Sirjan, Iran; d Expert of Khuzestan Water and Power Authority, Iran

**Keywords:** Optimization, Homashahr city, Water distribution network, Harris Hawks optimization algorithm

## Abstract

In this article applies the Harris Hawks Optimization Algorithm for optimization of the water distribution network of the Homashahr located in Iran for a period of one month (from 30 September 2018 to 30 October 2019). The utilized time-series data included water demand, reservoir storage. In this article, a model based on the Harris Hawks Optimization Algorithm (HHO) was developed for the optimization of the water distribution network. The analysis showed that the best solutions achieved by the Harris Hawks Optimization Algorithm (HHO) were 35,508 $. The results revealed that the HHO algorithm was well in the optimal design of water supply networks problem. At the end, about 12% of the optimization was done by this algorithm.

• In this article applied the Harris Hawks Optimization Algorithm for optimization of the water distribution network of the Homashahr located in Iran.
• The method presented in this article can be useful for managers of water and wastewater companies, water resource facilities and water distribution system managing director for optimal network design to reduce costs.
• The present algorithm performs better than the other algorithms in the discussion of the optimization of water distribution networks.

## Graphical abstract

**Figure:**
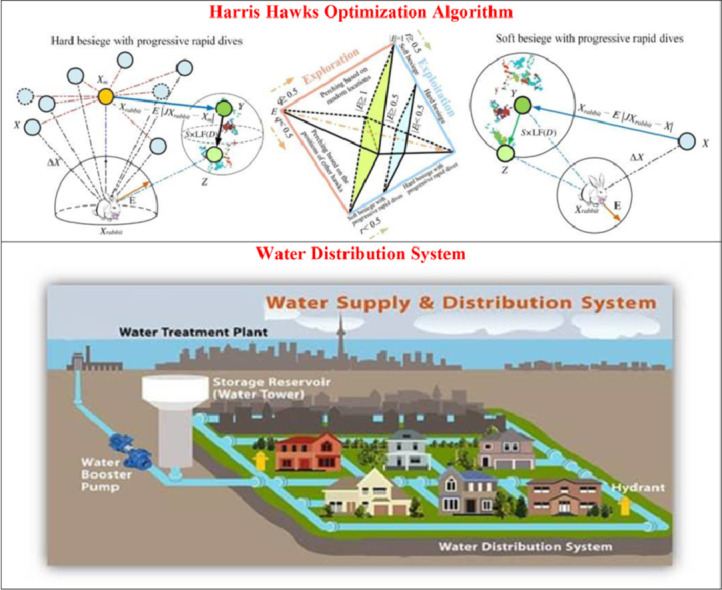

Specifications table

**Table:** 

Subject Area	Engineering
More specific subject area	*Optimization of problem using Harris Hawks Optimization Algorithm.*
Method name	*Harris Hawks Optimization Algorithm (HHO)*
Name and reference of original method	*Heidari, A. A., Mirjalili, S., Faris, H., Aljarah, I., Mafarja, M., & Chen, H. (2019). Harris hawks optimization: Algorithm and applications. Future generation computer systems, 97, 849–872.* https://doi.org/10.1016/j.future.2019.02.028.
Resource availability	*- Computer (Intel(R)CoreTMi7-7700 3.60-GHz 32 GB*
	*- MATLAB software*
	*- EPANET software*

## Literature review

During the last years, several Evolutionary Algorithms (EAs) including Water Cycle Algorithm (WCA) [1], [2], Moth–Flame Optimization (MFO) [3], [4], Mine Blast Algorithm (MBA) [5], Equilibrium Optimization Algorithm (EOA) [6], Butterfly Optimization Algorithm (BOA) [7], Grasshopper Optimization Algorithm (GOA) [8], Symbiotic Organism Search (SOS) [9], [10], [11], Moth-Swarm Algorithm (MSA) [12], [13], and Harris Hawks Algorithm (HHO) [14], [15] have been applied for solving different optimization problems [16].

The Harris Hawks Algorithm (HHO) is a one of the new meta-heuristic algorithms which be used in the different optimization subjects. So far, many researchers have been used this algorithm to solve some problems. Kurtuluş et al., (2020) was used the hybrid Harris hawks and simulated annealing algorithm (HHOSA) to optimize the design variables of the highway guardrails [17]. Yıldız et al., (2019) utilized the HHO in comparison with the grasshopper optimization algorithm (GOA) and the multi-verse optimization algorithm (MVO) for solving the manufacturing optimization problems [15]. Moayedi et al., (2019) compared the HHO related to the dragonfly algorithm to assess the bearing capacity of two-layer foundation soils [18]. Bao et al., (2019) introduced a novel hybrid HHO and differential evolution (DE) for color image multilevel thresholding segmentation [19]. Yıldız et al., (2019) compared some meta-heuristic algorithm including the HHO to optimize structural design of vehicle components [20]. Also, some other researches have been conducted in the field of engineering [21], [22], [23].

## Method description

In this paper, a model based on the Harris Hawks Algorithm (HHO) was developed for the optimization of the water distribution network (WDN) of the Homashahr located in Iran, which is effective in designing.

The HHO algorithm is coupled with the water distribution network analyze software, EPANET 2. After analyzing the model by EPANET 2, the pressure for each node and velocity of flow in each pipe are resulted then the values are compared to constraint limits.

The optimal design of water distribution systems is regarded as a problem at which the minimum cost is considered, and the diameter of pipes is considered as the main optimization parameter.

In the present paper, the time series Hydraulic dataset consists of Specifications of the nodes, pipe and, reservoir storage for a period of 1 year (2018). Also, all data is available in the Mendeley Data (https://data.mendeley.com/datasets/269dd3ywf2/draft?a=4b1596dc-f44e-4420–9b00-21c1ad32849b).

In the following, the methodology employed in this article, containing the WDN model, cost function, and optimization algorithms are explained in detail.

### The Harris Hawks algorithm

In this paper, the Harris Hawks Optimizer (HHO) algorithm as a nature-inspired optimization paradigm, is proposed to solve optimization problems by Heidari et al. in 2019 [14]. This algorithm is a population-based search that is implemented in three main phases.

In each iteration, search factors update its position using the three search operators for hunt (exploration phase), a transition from exploration to exploitation (extraction phase) and exploitation.

**Fig. 1:**
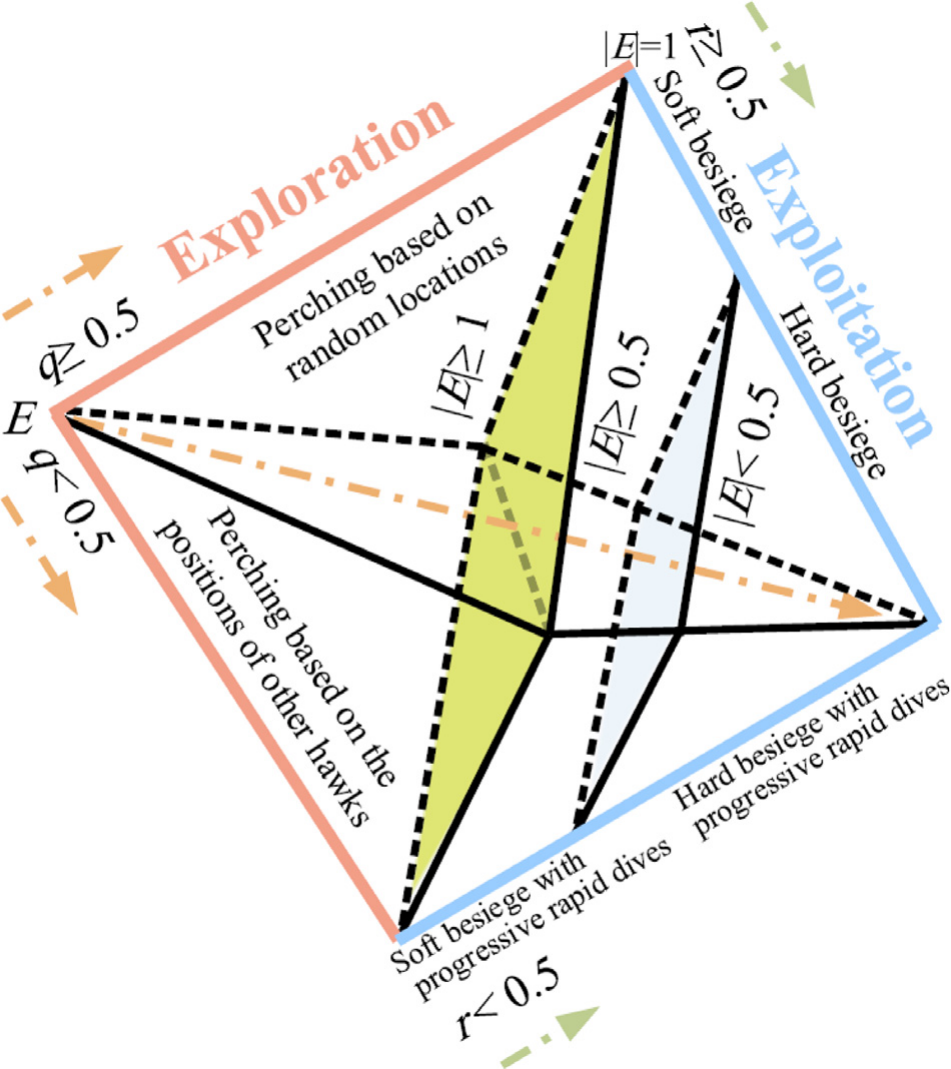
Different level of Harris Hawks optimization (Heidari et al. [14]).

The basic steps of the HHO algorithm are described as follows (Fig. 1):

### Exploration phase

The observation and discovery of prey by the Harris Hawks is performed at this step.

Usually, the Harris Hawks is on high trees to hunt prey.

A mathematical model is introduced to stimulate the searching process of Harris Hawks such as (1),

Where the Harris Hawks perch close to the hunt if *q*< 0.5 or perch on a random high trees if *q* ≥ 0.5, where q is a random number between 0 and 1.

(1) $$X\left(iter+1\right)=\left\{\begin{array}{c} X_{rand}\left(iter\right)-r_{1}|X_{rand}\left(iter\right)-2X_{r}\left(iter\right)...............if...q\geq 0.5\\ X_{rabit}\left(iter\right)-X_{m}\left(iter\right))-r_{3}\left(LB+r_{4}\left(UB-LB\right)\right)..if...q≺0.5 \end{array}\right\}$$

Where X_iter_ is the current location of candidate solution (hawks), X_rand_ is the randomly chosen hawk among the available population, X_rabit_ is the rabbit location, iter denotes the present iteration, r1, r2, r3, r4 and q are random numbers ranging in [0–1], LB and UB indicate the upper and lower values of the variables Xm displays the mean location of current hawks population and is computed as follows:

(2) $$X_{m}\left(iter\right)=\frac{1}{N}\sum_{i=1}^{N}X_{i}\left(iter\right),$$

In which X_i_ shows the location of each hawks and N represents the size of hawks’ population.

### Transition from exploration to exploitation phase

The HHO computes the running energy of the bait (E) using Eq. (3), where T is the maximum number of iteration and E_0_ is the initial energy. The running energy may be changed the exploration and exploitation.

(3) $$E=2E_{0}\left(1-\frac{iter}{T}\right)$$

The E_0_ varies randomly between [−1, 1] then E is changing from [−2, 2], but it decreases with the iteration increases as in (3). Therefore, if the fleeing energy of prey big (|E| ≥ 1) then the Harris Hawks are searching for fleeing prey (exploration stage), otherwise ((|E|< 1) then the Harris Hawks are trying to hunt the tired fleeing rabbits (exploitation stage).

### Exploitation phase

Based on escaping states of the rabbit and different tracking plans of the hawks, four possible solutions have obtained. These solutions have changed by variation of the running energy of the bait and the besiege strategies of the hawks.

The hawks may perform a soft or hard attack to hunt the bait from different path. The “r” describes the running chance of the bait. When the running chance of the bait is less than 0.5 (*r* < 0.5), the bait escape successfully, otherwise, the bait will be Caught. Moreover, when the running energy of the bait is more than 0.5 (|E| ≥0.5), the HHO performs a soft encircling maneuver, and otherwise, a hard attack is applied by the hawk. It is worth mentioning that a successful running of the bait, not only depends on the escaping energy, but also the running chance is effective parameter. Meanwhile, the process of trapping depends on both the hunt escaping and hawk besiege strategy [14].

### Optimization model formulation

There are several methods to optimize the water distribution network (WDN), which is defined in this paper the objective function of the cost reduction approach based on the reduction of the diameters by the permissible pressure in the nodes.

These problems are restrained by specified pressure head for nodes and sometimes specified velocity in pipe flow that can warranty service and secure flow with specified node demand. In this paper, the HHO algorithm is used for the optimization of water supply networks in order to minimize cost by changing pipe size. The equations for optimization can be written as: [24], [25], [26], [27]

(4) $$Minf_{\cos t}=\sum_{i=1}^{np}C_{D_{i}}.L_{i}$$

Subject to:

(5) $$\left\{\begin{array}{c} \sum Q_{in}-\sum Q_{out}=\sum Q_{e}\\ H_{j}\geq H_{j}^{\min} \end{array}\right\}$$

In which, C (_Di_) is the cost for unit length of diameter D for pipe i and L_i_ is the length of pipe i and n_p_ is the number of pipes used in the network. Continuity Equation is presented in Eq. (5) displays that the amount of water is being carried into the network should be consumed in nodal demands. There are different formulas for computing head loss and the Hazen Williams equation is one of the best known formulas regularly used for head loss calculation. The Equation used in this paper is Hazen-Williams formula as presented in Eq. (6):

(6) $$h_{f}=\sigma \frac{L}{C^{\beta }D^{\gamma }}Q^{\beta }$$

where, in SI system *σ* = 10.67, β = 1.85, ɣ= 4.87, Q is the flow (m^3^/s), C is the Hazen-Williams roughness coefficient which equal 150, D is the diameter of pipe (m), and L is pipe length (m), [25]. Excellent value for *σ*increases head loss and network needs larger diameter to deliver that amount of water because these can violate requirements for minimum pressure. Therefore, higher value for *σ* requires a more costly the water distribution network (WDN) design. The algorithm initiates the design process by selecting initial values for calculating design variables. Then, the algorithm checks the pressure head and velocity at each node and pipe and calculates the cost of the network. After processing a model, the pressure of each node is obtained and these values are compared to the allowable limits to compute the penalty functions as equations of (7) and (8):

(7) $$\left\{\begin{array}{c} H_{j}^{\min}\leq H_{j}\to \Delta_{j}=0\\ H_{j}\geq H_{j}^{\min}\to \Delta_{j}=\frac{H_{j}^{\min}-H_{j}}{H_{j}} \end{array}\right\},j=1,2,...,np$$

In this way, the objective function is defined by introducing the cost function as:

(8) $$F_{\cos t}=f_{\cos t}.\left(1+\varepsilon_{1}{\left(\sum \Delta_{j}\right)}^{\varepsilon_{2}}\right)$$

Here, the constant parameters are chosen considering the detection and operation rate of the search amplitude. ε_1_ is set to unity and ε_2_ choose in a way that it reduces the penalties and the values of the variables, as well. Thus, in the first steps of the search process, ε_2_ is set to 0.5 and ultimately increased to 1.05.

### Simulation

The HHO algorithm is coupled with the water distribution network analyze software, EPANET 2. After analyzing the model by EPANET 2, the pressure for each node and velocity of flow in each pipe are resulted then the values are compared to constraint limits. The software usage process implemented in MATLAB and optimization runs were carried out on a computer. A brief description of the stags that are taken for optimization of network is given below:

(1) Generate N CPs for starting analysis in MATLAB with random numbers. Each of the CPs is a possible combination of pipelines that indicates network to be solved.
(2) Compute the network cost for each population and sort them for analyzes mentioned in the HHO algorithm.
(3) Update the input file of the problem to be solved. In this type of optimization only pipe diameters are changed.
(4) Processes network using EPANET 2 for determining pipe flow velocities and node pressures.
(5) Generate penalty function and use it in determining the objective function.

### Case study

**Fig. 2:**
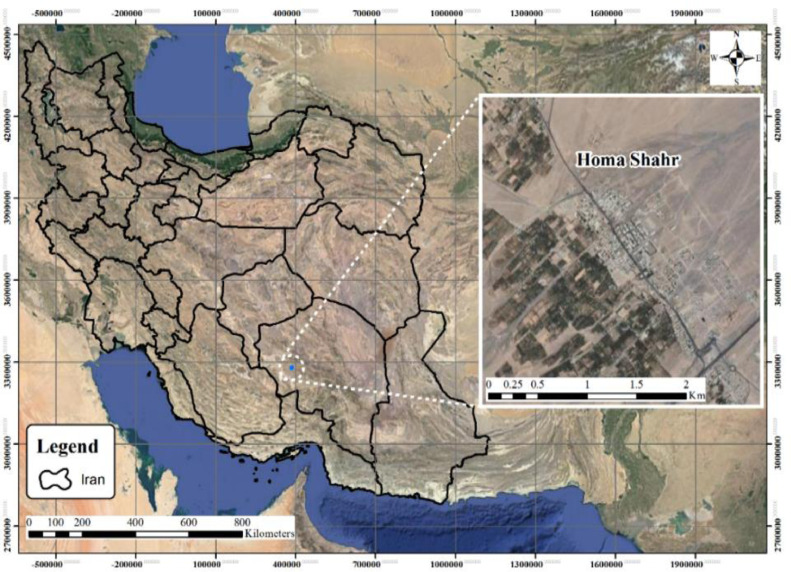
Location of the Homashahr-city in the Kerman Province (Southeastern of Iran).

**Fig. 3:**
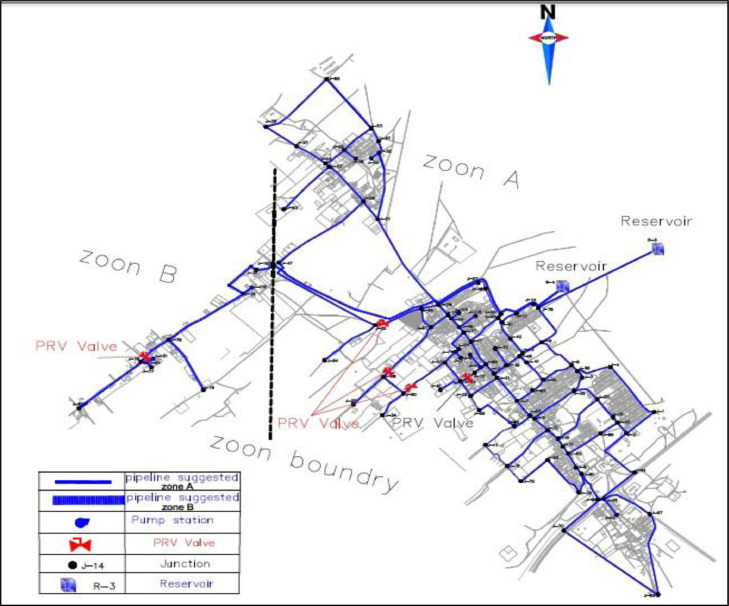
Homashahr network plan layout.

The Hamashahr network model, which was analyzed by Khalife et al. [28] in Sirjan, is shown in Fig. 2. As well as the Hamashahr network map is shown in Fig. 3. In 2018, Khalife et al. [28] used the Darwin Designer operator in Water Gems software, which was based on a genetic algorithm to optimize Hamashahr's water distribution network in Iran. While, in this article, the new Harris Hawks algorithm (2019) which has a stronger performance for optimization, is used for this purpose. This algorithm has also been used for the first time in optimizing the distribution network.

Another difference between this method and the previous method is writing code in MATLAB software, while the previous method of optimizing the water distribution network was done only with the Water Gems software itself.

The cost of available pipe sizes that are determined for consumption in this network are {63, 90, 110, 160, 200, 250, 315; in mm} that cost {0.95, 0.995, 1.066, 1.387, 1.445, 1.725, 2.137; in Dollar/meter}. The characteristics for pipes and nodes are presented and before optimizing in Table 1. The water required in this network is much higher than the accustomed demands for other ones, so for satisfying these demands, the maximum velocity limitation is set to 2 m/s. The pressure limit for nodes are minimum of 55 m and length of pipes are shown in Table 2 [29]. Brief description of the model in the following:

**Table 1:** Identification of the city's water distribution network Pipes before to the optimization.

Economic analysis before optimization			
Total Cost ($)	Cost per meter ($)	Length Pipe(m)	Diameter(mm)
9918	0.95	10,440	63
5968	0.995	5993	90
8475	1.066	7945	110
5872	1.387	4232	160
6286	1.445	4348	200
1128	1.725	654	250
3450	2.137	1614	315
41,097	Total

**Table 2:** Values of used algorithms parameters for problem.

HHO	parameter	Max iterations	Number of variables	Number of search agents
	Value	6000	3	100

Fig. 2 shows the location of the Homashahr city in the geographical map. Fig. 3 Shows that the Homashahr network plan layout.

**Fig. 4:**
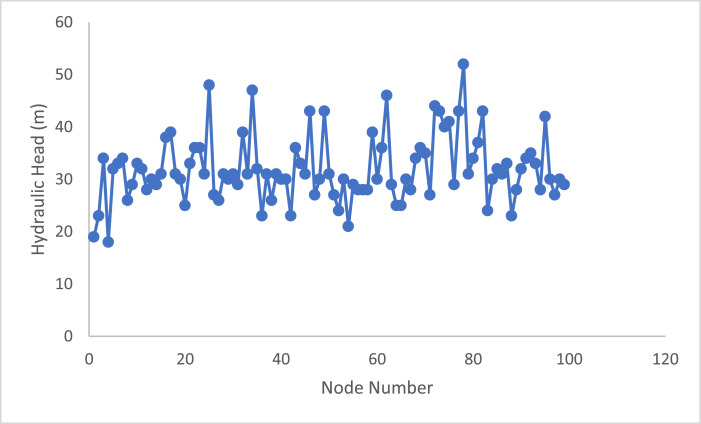
Nodal heads for Homashahr network.

Table 1 gives the main characteristics of the city's water distribution network Pipes before the optimization. Table 2 displays the Nodal Pressure head for the Homashahr network. Table 3 describes the objective Values of used algorithms parameters for the problem. Table 4 Show the Economic analysis after optimization with HHO. The Homashahr water distribution systems. Fig. 4 shows to converging in HHO Algorithm.

**Table 3:** Economic analysis after optimization with HHO.

Total Cost ($)	Cost per meter ($)	Length Pipe(m)	Diameter(mm)
2831	0.95	2980	63
3133	0.995	3149	90
6783	1.066	6363	110
8957	1.387	6458	160
5001	1.445	3461	200
3822	1.725	2216	250
4981	2.137	2331	315
35,508	Total

## Results and discussion

The analyses of this paper displayed that the best solution achieved the algorithm presented in this paper achieved the best solution answer for this network, which equals 35,508$ by 6000 iterations. It means convergence speed of this algorithm in achieving the minimum solution answer is good.

Constrain and nodal head pressure is presented in Fig. 4.

**Fig. 5:**
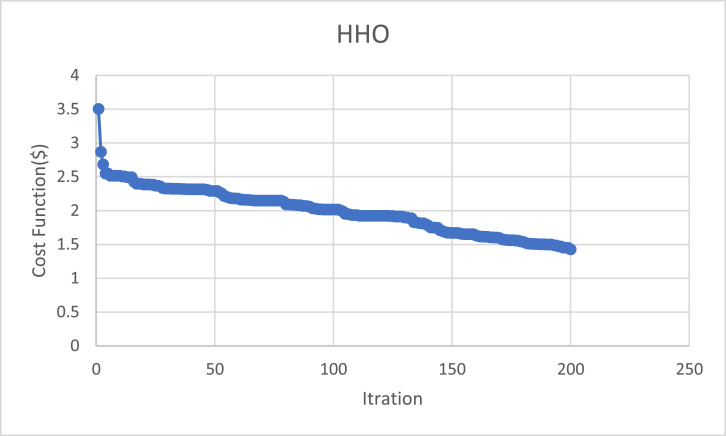
Converging in HHO Algorithm.

The speeds of the algorithm id good enough and it is shown in Fig. 5 that the best costs for the new algorithm can be obtained in lower iterations. The HHO algorithm reached 35,508 (13.6% away from best reached answer) after 600 iterations.

All analyses of this research for the HHO algorithm are presented in Tables 3 and Figs. 2 to 4.

## Declaration of Competing Interest

The authors declare that they have no known competing financial interests or personal relationships that could have appeared to influence the work reported in this paper.
